# Repeated cytoreductive surgery and Hyperthermic Intraperitoneal Chemotherapy in patients with peritoneal carcinomatosis: A retrospective cohort study

**DOI:** 10.1016/j.amsu.2021.102824

**Published:** 2021-09-15

**Authors:** C. Paasch, G. De Santo, H.N. Gamal-Eldin, M. Hünerbein

**Affiliations:** a University Hospital Brandenburg an der Havel, Brandenburg Medical University, Clinic for General and Visceral Surgery, Hochstraße 29, 14770, Brandenburg an der Havel, Germany; b Department of General Surgery, Oberhavel Kliniken Gransee, Meseberger Weg 12-13, 16775, Gransee, Brandenburg, Germany; c Center of Obesity and Metabolic Surgery, Helios Klinikum Berlin-Buch, Schwanebecker Chaussee 50, 13125, Berlin, Germany; d Department of Surgery, Oberhavel Klinik Oranienburg, Robert-Koch-Straße 2-12, 16515, Oranienburg, Germany

**Keywords:** HIPEC, Hyperthermic intraperitoneal chemotherapy, Colorectal cancer, Peritoneal carcinomatosis, Gastric cancer, Peritoneal mesothelioma

## Abstract

**Introduction:**

The prognosis of abdominal cancer with peritoneal carcinomatosis (PC) is poor. In literature, some authors described a repeated Cytoreductive Surgery (CRS) with Hyperthermic Intraperitoneal Chemotherapy (HIPEC) in patients with recurrent PC as feasible for overall survival improvement. Hence, we implemented this approach at our hospital and analyzed our cases.

**Methods:**

A unicentric retrospective observational study took place at the Helios hospital Berlin-Buch in 2020. The data of individuals who received a HIPEC in the time of 2007–2019 were extracted. The data were entered in the HIPEC database of the German Society of General and Visceral Surgery (StuDoQ|HIPEC, German society for general and visceral surgery). The primary objective was the overall survival after first HIPEC procedure.

**Results:**

A total of 292 data files from were extracted and 14 patients were identified as eligible for further analysis (7× colorectal, 3x gastric, 1× appendix cancer, 1× cancer of unknown primary, 1× Mesothelioma, 1× Pseudomyxoma peritonei). The mean age was 57 (8) years. The BMI was on average 23.5 (3.5) kg/m^2^. A total of 8 individuals were female and 6 male (6xASA-Score I, 8xASA-Score II). The initial Peritoneal Cancer Index (PCI) was on average 11.5 (9.1). The average overall survival after 1. HIPEC for colonic cancer was 74 months (n = 3; 43, 70 and 90 month), for gastric cancer 29 months (n = 2; 19 and 39 month) and for mesothelioma 44 months (n = 1).

**Conclusions:**

Based on our findings Repeated Cytoreductive Surgery with Hyperthermic Intraperitoneal Chemotherapy may improve overall survival of selected patients suffering from peritoneal carcinomatosis.

## Highlights

• The prognosis of abdominal cancer with peritoneal carcinomatosis is poor.
• In the past decade Cytoreductive Surgery with Hyperthermic Intraperitoneal Chemotherapy for peritoneal carcinomatosis has been implemented into daily routine.
• These approach may improve overall survival.

## 1 Introduction

Several malignant tumors lead to PC with poor overall survival [1].

In Western countries, approximately 15% of all cancer diagnoses have a colorectal origin. In up to 18% of these cases metachronous metastasis are diagnosed. The liver is the most common isolatic metastatic site [2] followed by the lungs, extra regional lymph nodes and the peritoneum, which is the second most common site of colorectal cancer recurrence (25–35%) [2,3]. With an overall survival of up to 12 months the prognosis of metastatic colorectal cancer is poor [3].

As the fifth most common cancer in the world gastric cancer accounts for 8.8% of cancer deaths yearly. PC occurs synchronous with the primary tumor in up to 14%–43% of cases. An overall survival of less than one year has been reported [4].

With approximately 800 new cases a year in the United States malignant peritoneal mesothelioma is a rare tumor entity. The overall survival has been estimated to be up to one year [5].

Also, malignances like the low-grade appendiceal mucinous neoplasms and pseudomyxoma peritonei may lead to poor overall survival due to PC [6].

To improve outcome of PC the CRS with HIPEC has been increasingly implemented into daily routine in the last two decades [7,8]. Subsequently, it has been demonstrated that these approach lead to an increased overall survival [8,9].

In literature, some authors also described a repeated CRP with HIPEC in patients with recurrent PC as feasible for further outcome improvement [10]. But the evidence remains low due to the lack prospective clinical trials.

The retrospective analysis at hand aimed to add more knowledge on that topic.

## 2 Methods

A unicentric retrospective observational study was conducted at the Helios hospital Berlin-Buch in 2020. The data of individuals who received a HIPEC were entered in the HIPEC database of the German Society of General and Visceral Surgery (StuDoQ|HIPEC, German society for general and visceral surgery). All patients prior subscribed a consent form. The data of individuals who received a HIPEC at the xxx in the time of 2007–2019 were extracted from the StuDoQ|HIPEC registry. Research Registry (researchregistry.com) has been used for study registration (ID: researchregistry7012).

The study at hand was conducted in accordance with the ethical standards of the Helsinki Declaration 1975 and with the publication guidelines of StuDoQ|HIPEC registry [11]. The study has been reported in line with the STROCSS criteria [12].

No funding has been received.

## 3 Objectives

The primary objective was the overall survival after first HIPEC procedure (months).

The secondary objectives were the cumulative length of hospital stay (days), Clavien-Dindo classification within 30 days after each HIPEC, recurrence-free survival (months), therapy-free survival after last HIPEC, mortality rate, chemotherapy prior to first HIPEC conduction.

### 3.1 Surgical approach

HIPEC was performed by the closed, semi-open or open technique using a Cisplatin, Mitomycin or 5-FU based regimen. After placement of Robinson drains (1× upper right, 1× upper left, 1× lower left, 1× lower right abdomen) and the temperature probe the chemotherapy was infused over 1 h with a temperature of 40°.

In cases of a normal renal function Cisplatin + Mitomycin C were used. Individuals who suffered from renal insufficiency or with a medical history of resistance to Cisplatin-based therapy received 5- Fluorouracil.

Considering possible chemotherapy-related complications such as an anastomotic leak HIPEC and CRS were conducted not simultaneously when an anastomosis was conducted. A period of approximately one week was considered as reasonable to perform HIPEC after CRS.

All procedures were conducted by one surgeon (work experience >25 years).

### 3.2 Patients’ selection criteria for repeated CRS + HIPEC

Patients’ selection criteria for repeated CRS + HIPEC are depicted in Fig. 2.

### 3.3 Statistics

In November 2020, the data was extracted from the StuDoQ|HIPEC registry of the German Society of General and Visceral Surgery. The data were put into a Microsoft© Excel data sheet. The descriptive analysis was done by using the built-in functions of Microsoft© Excel.

## 4 Results

**Fig. 1:**
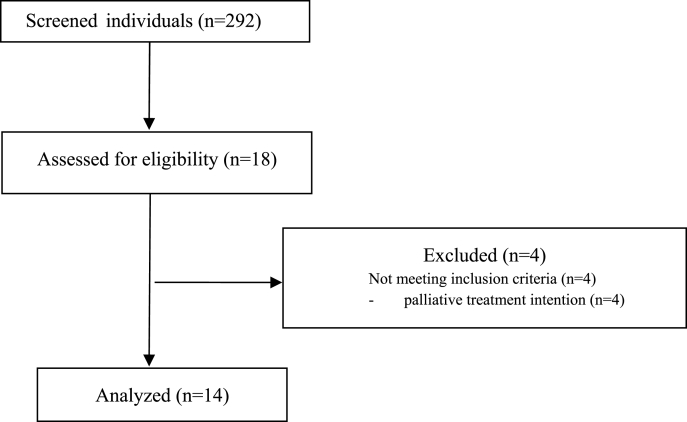
Flowchart for patients enrollment.

A total of 292 individual (♂ = 133, ♀ = 159; age: 64.3 ± 12.1) were analyzed, of whom 18 patients were identified as eligible. Due to palliative treatment intention 4 individuals were excluded from analysis. The data of 14 individuals were extracted from StuDoQ|HIPEC registry (Fig. 1). Table 1 provides detailed information on each patient.

**Fig. 2:**
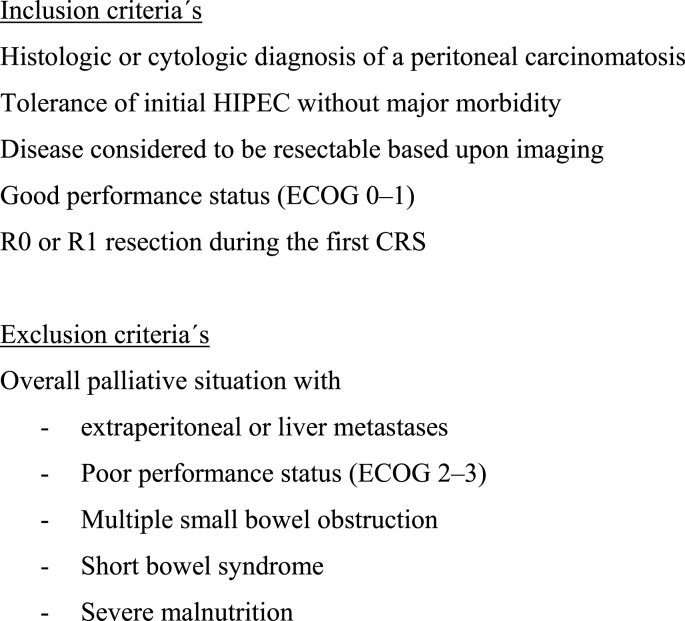
Patients' selection criteria for repeated CRS + HIPEC.

**Table 1:** Summarized data on patients receiving repeated CRS+HIPEC.

Patient	Gender	Age *years*	BMI *kg/m2*	ASA-Score *I–V*	Type of cancer	Initial PCI	Amount of HIPEC	HIPEC + CRC simultaneously *yes/no*	CT prior to 1. HIPEC *yes/no*	Duration 1. to 2. HIPEC *months*	Duration 2. to 3. HIPEC	CLOS *days*	OS *months*	RFS after 1. HIEPC *months*	TFS after last HIPEC *months*	
01	♀	65	25.0	II	Colonic cancer	7 (syn PC)	3 (Cisplatin + Mitomycin)	no	no	49	12	83 (IV)	70		NA	NA
02	♀	56	18.7	II	Colonic cancer	7 (syn PC)	3 (Cisplatin + Mitomycin)	no	no	20	14,6	36 (II)	NA/44	19	13	
03	♀	49	22.3	II	Colonic cancer	NA (syn. PC)	2 (5-FU)	no	no	23.7	–	49 (0)	NA/87	23	8	
04	♀	48	24.2	I	Colonic cancer	NA (met PC)	2 (Cisplatin + Mitomycin)	no	yes	8	–	37 (II)	NA/28	8	3	
05	♂	67	27.8	II	Colonic cancer	NA (syn PC)	2 (5-FU)	yes	no	7.8	–	46 (II)	43	8	6	
06	♂	65	24.5	I	Colonic cancer	NA (syn PC)	2 (Cisplatin + Mitomycin)	no	no	16	–	32 (I)	NA/16	3	NA	
07	♂	57	24.9	I	Colonic cancer	10 (syn PC)	2 (Cisplatin + Mitomycin, 5-FU 2. HIPEC)	yes	no	48.6	–	46 (II)	69	3	1	
08	♀	57	18.1	I	Gastric cancer	3 (syn PC)	3 (Cisplatin + Mitomycin)	no+	no	4.1	12.4	33 (II)	19	NA	NA	
09	♀	45	17.4	I	Gastric cancer	NA (syn PC)	2 (Cisplatin + Mitomycin)	no	yes	90	–	28 (NA)	NA/6	2	3	
10	♂	57	25.8	II	Gastric cancer	NA (syn PC)	2 (Cisplatin + Mitomycin)	no	no		665	–	28 (III)	39	21	7
11	♀	44	25.0	II	LAMN	NA (syn PC)	3 (Cisplatin + Mitomycin)	no+	no	331	371	58 (II)	NA/52	6	0	
12	♀	36	22.0	I	PMP	NA (syn PC)	2 (Cisplatin + Mitomycin)	no	no	766	–	57(II)	NA/114	25	0	
13	♂	56	27.7	II	Mesothelioma	11 (syn PC)	2 (Cisplatin + Mitomycin)	no	no	192	–	45 (II)	44	3	39	
14	♂	34	25.0	II	CUP	31 (syn PC)	2 (Cisplatin + Mitomycin)	no	no	1106	–	25 (NA)	NA/162	NA	NA	

BMI Body mass index; CLOS cumulative length of hospital stay; CRC cytoreductive surgery; CT chemotherapy.

LAMN Low-grade appendiceal mucinous neoplasm; PC peritoneal carcinosis; PCI peritoneal carcinosis index.

Met metachronous; NA not applied; NA/months not applied/patient is alive; OS overall survival; PMP pseudomyxoma peritonei.

RFS recurrence.free survival; Syn synchronous; TFS therapy-free survival; + no repeated CRS.

() CDC Clavien-Dindo-classification during hospital stay; CUP cancer of unknown primary.

The mean age was 57 (8) years. The BMI was on average 23.5 (3.5) kg/m^2^. A total of 8 individuals were female and 6 male. An ASA-Score of I was determinate in 6 and of II in 8 cases (Table 2).

**Table 2:** Baseline characteristics.

Variable		Study group n = 14
Age	years	57 (8)
Gender	male	6
	female	8
ASA preoperative	I	6
	II	8
	III-V	0
BMI	kg/m2	23.5 (3.5)

ASA = American Society of Anesthesiologists physical status classification; BMI Body Mass Index.

Continuous measurements are presented as mean (SD).

In terms of perioperative data the cumulative length of hospital stay was 43 (15.1) days. A total of 7 individuals suffered from colonic cancer. In 5 cases a HIPEC was conducted twice and in 3 cases 3 times. Gastric cancer was diagnosed in 3 patients. In 2 cases a HIPEC was performed twice and in one case 3 times. A Pseudomyxoma peritonei, a low-grade appendiceal mucinous neoplasm, a mesothelioma and a cancer of unknown primary occurred singularly. Each of these 4 patients received a HIPEC twice.

The initial Peritoneal Cancer Index (PCI) was on average 11.5 (9.1). In 8 cases no data on PCI were available. In 13 cases the PC occurred synchronously and in one case metachronously (Table 3).

**Table 3:** Perioperative data I.

Variable		Study group *n* = *14*	Amount of HIPEC conduction
Cumulative LOS	*days*	43 (15.1)	
Clavien-Dindo classification^b^	0	1	
	*I*	1	
	*II*	8	
	*III*	1	
	*IV*	1	
	*NA*	2	
Type of malignancy			
	*colonic cancer*	7 (50.0%)	5 × 2; 2 × 3
	*gastric cancer*	3 (21.5%)	2 × 2; 1 × 3
	*pseudomyxoma peritonei*	1 (7.1%)	1 × 2
	*low-grade appendiceal mucinous neoplasm*	1 (7.1%)	1 × 2
	*mesothelioma*	1 (7.1%)	1 × 2
	*CUP syndrom*	1 (7.1%)	1 × 2
Type of peritoneal carcinosis	synchronous	13 (92.9%)	
	metachronous	1 (7.1%)	
Initial PCI Score	11.5 (9.1)^a^		

Continuous measurements are presented as mean (SD).

LOS length of hospital stay; NA not applied.

PCI Peritoneal Cancer Index.

a In 8 cases no available initial PCI Score.

b Within 30 days after each HIPEC.

Regarding information on survival after repetitive HIPEC conduction and CRS a death rate of 35.7% (n = 5) was detected. A total of 7 individuals did not reached overall survival (7 patients still alive, Table 1). The average overall survival after 1. HIPEC for colonic cancer was 74.4 months (n = 3; 43, 70 and 90 month), for gastric cancer 29 months (n = 2; 19 and 39 month) and for mesothelioma 44 months (n = 1; Table 4).

**Table 4:** Survival after repetitive HIPEC conduction.

Variable		Study group n = 14
Death rate	*n*	5 (35.7%)^c^
Overall survival	*months*	
colon cancer		74.4
gastric cancer		29
mesothelioma		44
Recurrence-free survival after 1. HIPEC^c^	*months*	11 (8.6)^a^
Therapy-free survival after last HIPEC^c^	*months*	8 (11.6)^b^

All detailed data are depicted in Table 1.

a Not applied in 3 cases.

b Not applied in 4 cases.

c Recurrence- and Therapy-free survival has been summarized.

In terms of postoperative complication within 30 days of surgery. One individual has a CDC-Classification of 0, one of I, 8 of II, one of III, one of IV. In 2 cases the information was not applied (Table 1, Table 3).

Two individuals received chemotherapy prior to repeated HIPEC (Table 1, Patient 4 and 9).

## 5 Discussion

The PC of gastric, colonic and appendix cancer as well as peritoneal mesothelioma lead to poor overall survival. Therefore, next to single conduction several authors published their experience on repeated CRS and HIPEC [9]. To that, Vassos et al. (2016) performed a retrospective analysis of 6 cases and a review of literature on that topic. A total of 11 studies were reviewed with a cumulative sample size of 343. The individuals suffered from various malignant diseases with PC. A 5-year survival rate after 1. HIPEC was on average up to 30% [10]. Chua et al. (2013) and Votanopoulos et al. (2012) reported the highest sample sizes [13,14]. A total of 79 patients with an overall survival of 48-month were reported by Chua et al.. Their patients suffered from pseudomyxoma, appendiceal carcinoma, small bowel carcinoma, ovarian cancer and hepatocellular carcinoma [10,13]. Votanopoulos et al. (2012) published the data of 62 individuals. These patients had appendiceal carcinoma, colorectal cancer, mesothelioma, ovarian cancer, gastric cancer, GIST, gallbladder carcinoma, small bowel carcinoma, leiomyosarcoma and urachal carcinoma. A 32-month overall survival was stated [15]. We revealed comparative results. The patients analyzed in our study survived on average 74.4 months with colonic cancer and with gastric cancer 24 months. Nevertheless, due to different inclusion criteria and endpoints these studies are only to distinct degree comparable to each other. Prospective trials are needed on that topic.

To avoid extended therapy the usefulness of repeated CRS and HIPEC must be further discussed. To that, we reviewed the literature to identify suitable publications to compare overall survival for each tumor entity with our own results.

In terms of colonic cancer, we revealed an overall survival of 74.4 months among our patients (n = 3, average PCI: 8,5, in one case not applied; Table 1). Exemplary Hallam et al. (2019) performed a meta-analysis on 24 studies including 3128 patients. The authors excluded studies on repeated HIPEC and CRS. The patients were treated with single HIPEC and CRS due to colorectal cancer with PC. The authors stated an overall survival across all studies of 32 months (12.1–52) [16]. A total of 4 individuals in our study are still alive (16, 28, 44 and 87 months). The overall survival among our patients was more than two times higher in comparison to Hallams findings. But their meta-analysis also included rectal cancer and the reviewed studies may choose different approaches to measure overall survival and had different initial PCI-scores. Hence, our results may allow the assumption that repeated HIPEC and CRS improve overall survival. Further trials are mandatory.

A total of 3 individuals with gastric cancer and PC received repeated CRS and HIPEC in our study. We revealed an overall survival of 29 months (n = 2; average PCI: 3; Table 1). In comparison to that, Gill et al. (2011) published a systematic review on single HIPEC and CRS conduction (7 prospective and 3 retrospective studies; n = 441 patients). The authors stated an overall survival of 15 months [17].

One male patient among our cohort suffered from a mesothelioma with PC. He survived 44 months (initial PCI: 11). In comparison to that, Cashin et al. (2019) published results from the Swedish HIPEC registry and the Swedish National Cancer Registry. An overall survival of 15 months (n = 6) was revealed [18]. In addition, Ali et al. (2020) reported an overall survival of 40,8 months, when conducting a randomized clinical trial among 46 individuals who suffered from a mesothelioma with PC. A single HIPEC with CRS was conducted [5].

In terms of appendix carcinoma, CUP and pseudomyxoma peritonei only one patient was operated on with repeated HIPEC with CRS. All three patients are fortunately still alive with stable disease and did not reach overall survival (Table 1) for further comparative analysis.

Summarized, we retrospectively analyzed only a small cohort of patients and compared our results with historic data. In terms of colonic and gastric cancer with PC repeated HIPEC and CRS may improve overall survival.

Mostly younger patients (<60) in good health condition were analyzed in the mentioned studies [10,16,19]. Single or repeated HIPEC and CRS may improve overall survival in selected patients. In contrast, Arslan et al. (2018) performed a retrospective analysis on 100 individuals, who were operated on with single HIPEC and CRS due to colorectal cancer with PC. The authors revealed that this approach offer comparable oncologic outcome in selected elderly individuals without increased postoperative morbidity [19].

The retrospective study design, the small sample size and the lack of initial PCI-scores in 8 cases must be considered as a study limitation. Moreover, a retrospective analysis focusing on one disease would be favorable. The patients on average aged 57 (8) in this analysis had only an ASA-Score of I or II. This can also be considered as a selection bias. To state comparable 5-year survival rates a long-term follow-up will be conducted on the individuals analyzed in the study at hand.

## 6 Conclusion

Repeated Cytoreductive Surgery with Hyperthermic Intraperitoneal Chemotherapy may improve overall survival of selected patients suffering from peritoneal carcinomatosis. Randomized trials are mandatory to confirm our findings.

## Ethical approval

All patients gave informed consent.

## Sources of funding

All authors have no source of funding.

## Trial registry number

No funding has been received. Data from the HIPEC database of the German Society of General and Visceral Surgery (StuDoQ|HIPEC, German society for general and visceral surgery) were used. All patients gave their consent.

Research Registry (researchregistry.com) has been used for study registration (ID: researchregistry7012).

## Author contributions

Dr. med. Christoph Paasch (corresponding author):

Contribution to the paper: author, data collection, data analysis and interpretation, writing the paper, examination and treatment of the patient.

Dr. Gianluca De Santo (co-author):

Contribution to the paper: data analysis, examination and treatment of the patient.

Herr Gamal-Eldin (co-author):

Contribution to the paper: data analysis, examination and treatment of the patient.

Prof. Dr. Michael Hünerbein (senior-author):

Contribution to the paper: data analysis, examination and surgical treatment of the patient.

## Guarantor

Dr. med. Christoph Paasch.

## Declaration of competing interest

None.
